# A Continuous Statistical Phasing Framework for the Analysis of Forensic Mitochondrial DNA Mixtures

**DOI:** 10.3390/genes12020128

**Published:** 2021-01-20

**Authors:** Utpal Smart, Jennifer Churchill Cihlar, Sammed N. Mandape, Melissa Muenzler, Jonathan L. King, Bruce Budowle, August E. Woerner

**Affiliations:** 1 Center for Human Identification, University of North Texas Health Science Center, 3500 Camp, Bowie Blvd., Fort Worth, TX 76107, USA; utpal.smart@unthsc.edu (U.S.); jennifer.cihlar@unthsc.edu (J.C.C.); sammed.mandape@unthsc.edu (S.N.M.); melissa.muenzler@unthsc.edu (M.M.); jonathan.king@unthsc.edu (J.L.K.); Bruce.Budowle@unthsc.edu (B.B.); 2 Department of Microbiology, Immunology and Genetics, University of North Texas Health Science Center, 3500 Camp Bowie Blvd., Fort Worth, TX 76107, USA

**Keywords:** bayesian inference, bioinformatics, computational phasing, DEploid, population genomics, forensic genetics, Ion Torrent, massively parallel sequencing, mtDNA mixture deconvolution, R

## Abstract

Despite the benefits of quantitative data generated by massively parallel sequencing, resolving mitotypes from mixtures occurring in certain ratios remains challenging. In this study, a bioinformatic mixture deconvolution method centered on population-based phasing was developed and validated. The method was first tested on 270 in silico two-person mixtures varying in mixture proportions. An assortment of external reference panels containing information on haplotypic variation (from similar and different haplogroups) was leveraged to assess the effect of panel composition on phasing accuracy. Building on these simulations, mitochondrial genomes from the Human Mitochondrial DataBase were sourced to populate the panels and key parameter values were identified by deconvolving an additional 7290 in silico two-person mixtures. Finally, employing an optimized reference panel and phasing parameters, the approach was validated with in vitro two-person mixtures with differing proportions. Deconvolution was most accurate when the haplotypes in the mixture were similar to haplotypes present in the reference panel and when the mixture ratios were neither highly imbalanced nor subequal (e.g., 4:1). Overall, errors in haplotype estimation were largely bounded by the accuracy of the mixture’s genotype results. The proposed framework is the first available approach that automates the reconstruction of complete individual mitotypes from mixtures, even in ratios that have traditionally been considered problematic.

## 1. Introduction

One of the most challenging areas within forensic genetics is the interpretation of mixed DNA samples, especially those characterized by mitochondrial DNA (mtDNA) [1,2,3,4]. In the broadest sense, the presence of multiple types of organellar genomes within an individual or cell (i.e., heteroplasmy) and the highly unlikely and controversial phenomenon of mitochondrial paternal leakage [5,6] could classify as types of DNA mixture [4,7,8]. However, generally, forensic mixtures refer to a forensic sample comprising multiple donors. Within this context, the objective of deconvolving these mixtures is to estimate the number of contributors and tease apart their individual contributions, typically relying on relative proportions of the representative profiles. The target loci of choice for interpreting genetic mixtures have traditionally been short tandem repeats (STRs; otherwise also known as microsatellites) [9]. However, to handle samples with low quality nuclear DNA, several studies have proposed and explored the alternative of performing mixture interpretation with mtDNA [3,4,10,11,12].

For almost three decades, mtDNA has played a pivotal role in forensic genetics and related casework [8,10,13,14,15]. The main appeal of mtDNA as a forensic marker stems from certain unique properties of the mitochondrial genome (mtGenome) that are not exhibited by its nuclear counterpart. For example, mtDNA exists in high copy number (anywhere from a 100–1000 copies per cell), making it easier to extract and amplify from forensic samples that can be degraded and contain minute quantities of nuclear DNA [8,16]. Furthermore, being a haploid marker implies that only a single haplotype is added to the mixture by each contributor, thus significantly reducing the complexity of the interpretation. Other valuable properties of mtDNA include a high mutation rate (>10× relative to nuclear DNA), lack of recombination, and a well-characterized phylogeny [8,10,17,18,19]. Sanger sequence data, the primary forensic method for analysis of mtDNA, have limitations in deconvolving mtDNA mixtures because the data are not quantitative and assignment of observed variants to a donor is difficult [4,20,21]. Given these limitations of Sanger sequence data, the interpretation of mixed mtDNA forensic samples was seldom considered, until the advent of massively parallel sequencing (MPS) [3,20,22]. The introduction of MPS has made the use of mtDNA as a forensic tool more tractable by facilitating the sequencing of full mtGenomes and generating large amounts of quantitative genetic data in general [3,4,20,22,23]. Linkage information and related quantitative data allow for potential reconstruction of individual haplotypes and detection of novel and low-frequency variation [3,4,10,24].

However, deconvolving MPS-derived data for mtDNA mixtures comes with its own distinctive set of challenges. While quantitative information (e.g., read depth) facilitates deconvolution of certain types of mixtures, sequence reads produced by MPS tend to be too short to allow for the accurate estimation of discrete haplotypes. This length constraint is especially relevant given that forensic samples can be highly degraded [4,10]. Moreover, studies have shown that quantitative data are most readily interpreted (i.e., deconvolved) in two-person mixtures that are dissimilar in proportion [3,10,25]. Mixtures that occur at or near equal proportions (e.g., 50/50) or that involve three or more individuals are more difficult to interpret. If the main aim is to derive mitotypes of contributors, starkly disparate or imbalanced mixtures (e.g., 50:1) can also be problematic, as it can be computationally difficult to discern a minor contributor from noise [3,4,10,24].

Several computational tools have been created to tackle the challenges presented by mixed DNA profiles (see [26,27,28,29] for a comprehensive list of forensic mixture deconvolution software), all of which can be divided into two broad strategies based on the underlying mathematical model, namely continuous methods and semi-continuous methods. The former make use of both quantitative (peak heights or read depth) and qualitative (allele calls) information, while the latter only utilize qualitative data [27,28,30,31,32,33]. Nonetheless, a large majority of programs employing these models are made for autosomal data and only a couple of approaches have been dedicated to automated deconvolution of mtDNA mixtures. The software mixemt [10], for example, exploits the well-annotated phylogeny of human mtGenomes to approximate the haplogroups of donors in a mixture. Briefly, the software searches each mapped read for variants described in the public database Phylotree [34] and calculates the probability of the read originating from each haplogroup based on its estimated frequency. To this end, the software employs an iterative process to approximate the maximum likelihood function, known as the expectation maximization algorithm (EM [35]), which helps allocate the most likely haplogroup(s). Several studies have shown that utilizing available information on human mtDNA variation can help with the separation of some mixtures (i.e., 50/50 or 99/1), since it allows for the phylogenetic consignment of observed variants to specific haplogroups [10,19,24,36]. Nonetheless, according to personal experience with PCR data, this implementation can be time-consuming and resource-intensive (~20 gigabytes of memory and run times in excess of 48 h for two-person mtDNA mixtures). Moreover, since the software works on reads, it carries the risk of introducing noise factors such as NUMTs (i.e., nuclear mitochondrial insertions; however see [37,38]) More importantly, while this approach returns the most likely haplogroup(s), it only yields possible sequences of the deconvolved individual haplotypes under important assumptions. The software depends on common variants to attribute sequences to individual donors. This reliance implies that it would only be able to produce complete contributor haplotypes if (1) the haplogroup of the single-source mitotypes are considerably distinct (i.e., belong to considerably dissimilar haplogroups) and (2) comprise long DNA fragments. If these assumptions are not met, mixemt is restricted to inferring haplogroups, an ability that limits its value and is less than ideal for human identification, which hinges on the accurate estimation of haplotypes [4].

Another promising analytical approach to deconvolving mtDNA mixtures involves using phasing algorithms. Phasing refers to the estimation of haplotype data from genotypic data [39,40]. In other words, it helps to identify alleles that occur together on the same chromosome (i.e., linked alleles). This is useful, since genotypic data (other than those variants contained within a short fragment or amplicon) do not allow for the direct observation of which of the two parental chromosomes (i.e., haplotypes of a diploid genome) a specific allele resides in. In the context of mixtures of haploid genetic material such as mtDNA, each contributor can be considered as a parent and their combined DNA as the genotype. Phasing of mixed mtDNA can, thus, enable estimation of the individual haplotypes of each donor. In the approach of Stephens et al. [41], this strategy records the position of each single-nucleotide polymorphism (SNP) as having one of four states: one copy of the alternative (i.e., non-reference) allele and one of the reference (henceforth referred to as He); two copies of the reference nucleotide (henceforth referred to as HoR); two copies of the (same) alternative allele (henceforth referred to as HoA); and infrequently, one copy each of two different alternative nucleotides.

Given that experimental phasing (laboratory-based) methodologies are generally more cumbersome and expensive [39,42,43], a host of statistical phasing (computation-based) software are currently available (e.g., PHASE [41], SHAPEIT [44], BEAGLE [40], IMPUTE2 [45]). In general, these programs depend on linkage disequilibrium (LD) information within the data and occasionally on a reference panel (i.e., database of haplotypes that is used as a reference in order to increase phasing accuracy) [39,40,42,46,47]. However, all of these methods are designed for (single-source) diploid data, and therefore are not suitable for mtDNA mixture deconvolution. This is primarily because, unlike genotypes consisting of diploid DNA, haploid mixtures can be polygenomic (i.e., comprise of haplotypes of more than two individuals) and the genetic material of each contributor can be present in varying proportions [48].

Recent advances in the computational epidemiology of malaria may provide an expedient framework for the phasing of mtDNA mixtures in forensic samples. Multiple programs have been devised with the purpose of identifying different haploid strains of the parasitic protozoan *Plasmodium falciparum* and the proportions in which they occur within the host [48,49,50]. Out of these, DEploid (version 0.5.2) is the only software that is able to deconvolve strain haplotypes [48]. DEploid uses a Markov chain Monte Carlo (MCMC) process [37] to deconvolve mixtures of different strains (i.e., haplotypes) of *P. falciparum* by factoring in their numbers, relative abundance, and allelic states in order to estimate the number of different genetic types present in the mixture, quantify the proportion of each haplotype, and recover their nucleotide sequences. The posterior probabilities of each of these factors is conditioned on a read error rate and prior information on allele frequencies provided by a reference panel. The number of haplotypes present in the mixture is derived by starting a MCMC chain, with the number of strains ranging from 1 to 5, in order to reflect the maximum number of haplotypes that can be estimated with confidence. The prior is set up to assure that only haplotypes that are present in a substantial proportion are assessed. The relative proportion of each haplotype is estimated by applying the Metropolis–Hastings implementation of MCMC [51,52], leveraging information on the disparities of allele frequency rates within samples. The haplotypes are estimated from the reference panel within a Li and Stephens [41,53,54] framework. A Gibbs sampler [55] updates the allelic state of the haplotype from the posterior by tracing a path through the reference panel. The recombination rate parameter, which would not be used for mtDNA analysis (which can be set to 0.0 for mtDNA analyses), permits swapping between haplotypes in the panel, while the mis-copying rate parameter allows for mixed haplotypes to differ from those in the reference panel.

DEploid is reported to be comparable to diploid phasing methods when it comes to estimation of mixture proportions, while being considerably better at haplotype inferences [48]. For example, DEploid resulted in a notably lower switch error rate (the percentage of consecutive pairs of heterozygote loci in an individual phased incorrectly relative to each other [40,43,56]) and genotype error rate (discordance between the individual haplotypes and their corresponding true haplotypes [57]) relative to the other programs. This performance, combined with the fact that it is one of the few software programs that reconstructs mixed mtDNA haplotypes, makes DEploid an attractive tool to explore for the deconvolution of mtDNA mixtures. 

In this study, the utility of DEploid for deconvolving human mtDNA mixtures within a forensics context was investigated. To this end, a custom bioinformatic pipeline in the R statistical computing software [58] was created around the core DEploid phasing algorithm. This set-up systematically appraised the performance of the program, focusing on two-person mtDNA mixtures and reference databases of varying sizes and phylogenetic affinities. Within this context, real-world data were used to make computer-generated mixtures, and key software parameters were methodically manipulated to assess the accuracy of phasing. Finally, in vitro samples were used to validate the approach. In addition to determining if the software is able to correctly estimate the numbers of genomic donors in the mixture, their relative proportions, and their true haplotype structure, the broader research questions driving this study were:

1. How does the algorithm perform with rare (i.e., low frequency) variants?
2. What kind of effects do increases of the size (thereby population haplotype frequency information) and quality (presence of similar haplotypes) of the panel have on phasing accuracy?
3. Which statistical phasing strategies can be employed to better handle mixture ratios that have been traditionally harder to deconvolve (e.g., 1:1 and 50:1)?

## 2. Materials and Methods

### 2.1. Simulations with In Silico Mixtures

#### 2.1.1. Outline of Data and Bioinformatic Approach

Given the number of variables involved in high-throughput sequencing (sequence error, strand bias, and allele drop-out, amongst others) and their stochastic nature, testing of our algorithm was first performed in a simplified set-up. While the sequence data itself were real, the mixtures were first generated in silico to assure that genotype data for the mixed samples and the allelic proportions were accurate. This approach provided more confidence that the results being observed reflected the performance of the algorithm instead of being confounded by the noise present in real mixture data.

The required pieces of information for the core algorithm include counts (i.e., read depth) of alternative and reference alleles in the mixture, a reference panel consisting of mitotypes from a population allied to haplotypes in the mixture, and population-level allele frequency data. Accordingly, in silico mixtures were created using full mtGenome data from a subset of 10 haplotypes from 521 samples generated by [21]. Even though these individuals all belonged to a Brazilian Native American population, they spanned a total of 8 distinct haplogroups (Table S1). The genomes were entered into the pipeline as tab-separated files, which contain information on mitogenomic sites that vary from the revised Cambridge reference sequence (rCRS [59]) in their sampled haplotypes (henceforth referred to as EMPOP files, based on the format of the European DNA Profiling [EDNAP] Group’s mitochondrial DNA population database project) [60]. Two-person in silico mixtures with all available pairs of haplotypes using ratios ranging between 1:1 and 50:1 were then created from the haplotype data. DEploid was designed for use with shotgun sequencing data and requires input of the number of reads that support each allele call. To adapt this requirement to PCR-amplified data, idealized ratios (e.g., 1:9) were normalized into read counts, assuming a total read depth of 100× (e.g., 10:90 as per the 1:9 example). This total read count attempts to replicate 100 independent reads. Given that the software does not handle multi-allelic sites (i.e., infinite sites violations [61]) and insertions-deletions (indels), the data were filtered for any loci with more than two alleles and sites that represented indels before creating the panel and mixtures.

Freely available mtGenomes from the Human Mitochondrial DataBase (HmtDB [62,63]) website (https://www.hmtdb.uniba.it/) were used to construct comprehensive reference panels. These data consist of ~44 k mtGenomes from continent-specific groups, namely European, African, Asian, Oceanian, and South Asian groups. These data are available as FASTA files for individual mtGenomes with SNP calls. However, since these calls are not generated using forensic standards, it was necessary to apply identical scaffolds to both the HmtDB and our MPS data. To this end, first each amplicon of every single mtGenome was aligned to the rCRS in an effort to extract sites that varied from the reference genome. To reduce computation time, only those amplicons that were unique were chosen. The resulting set of amplicons was converted from FASTA to FASTQ (all base quality scores were set to the highest value, i.e., Q_20_) and finally to the bam file format using custom UNIX and R scripts. The bam files were then run through the software Converge version 2.0 (ThermoFisher Inc., Waltham, CA, USA) to realign and call variants so as to produce the EMPOP files (for more details on the preprocessing of these data, see [37]).

Downstream analyses included the validation of phased haplotypes. Even though various measures to estimate statistical phasing accuracy are regularly used in the literature (namely haplotype accuracy, switch error, and imputation accuracy), using just one measure that is apposite to the specific study is typically adequate [39,40]. Haplotype accuracy (or phasing accuracy) is defined as the proportion of haplotypes that are inferred correctly across the region of interest [39]. This metric was approximated by comparing the phased haplotype to the true haplotype using the Hamming distance [64]. Generally, this process involves calculating the total number of nucleotides that differ between the two haplotypes *a* and *b*, which can be expressed as *d*(*a*, *b*). However, considering two-person mixtures, the Hamming distance was computed between two sets of haplotypes—one containing the two true haplotypes (*A*, *B*) and the other containing the pair of estimated haplotypes (*a*, *b*). This first required the computation of *d* (*A*, *a*), *d* (*A*, *b*), *d* (*B*, *a*), and *d* (*B*, *b*). The total pairwise difference between the true and estimated haplotypes was then acquired by choosing between the smaller of the two Hamming distances *d*(*A*, *a*) + *d*(*B*, *b*) and *d*(*A*, *b*) + *d*(*B*, *a*). A total pairwise difference of zero, for example, would indicate 100% phasing accuracy for both mitotypes, with progressively higher values reflecting a corresponding drop in haplotype estimation accuracy. Additionally, deconvolution accuracy was defined as the percentage of mixtures where both donors have a Hamming distance of zero between true and estimated haplotypes. Henceforth, if a mixture or a haplotype is said to be “phased accurately or correctly”, it indicates 100% accuracy.

#### 2.1.2. Investigating the Effect of Panel Composition on Phasing Accuracy

**Figure 1:**
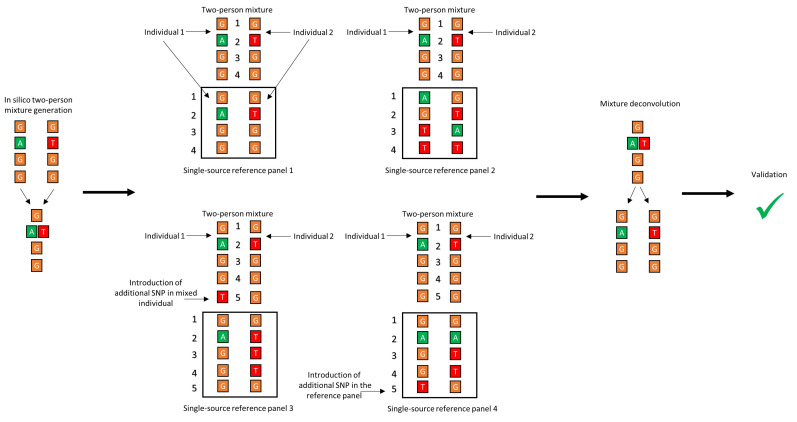
Graphical representation of the differences of the 4 reference panels used in the mixture deconvolution simulations.

The authors of DEploid (and multiple other studies [39,40,47,65,66]) emphasized the need for an appropriate reference panel as the single most important factor in increasing the accuracy of haplotype inference. However, panel files from an empirical setting are unlikely to comprehensively characterize the haplotype diversity of the population(s) of interest. To better understand how the quality of the panel file influenced the accuracy of haplotype inference, the bioinformatic pipeline was first tested using four different panel types (Figure 1):

1. Panel 1: Replicating an idealized scenario, this panel included both of the single-source haplotypes included in the mixture.
2. Panel 2: Using a hold-out cross validation (CV) approach [67,68], namely the hold-two-out CV, this panel was made by leaving out the two haplotypes that were used to create the mixture during each round of deconvolution. The hold-out CV allows validation of the performance of the core algorithm, as well as mimicking of real-world forensic data, whereby haplotype information for both donors is unlikely to be included in the reference database.
3. Panel 3: Reproduction of the presence of a closely related haplotype within the reference data, which was done by creating “ancestral” and “derived” haplotypes that differed from one another by one SNP. This difference was introduced in one of the haplotypes in the mixture while keeping the original sequence in the panel file. The other haplotype was excluded from the panel.
4. Panel 4: Same as above for panel 3 except this time one SNP was introduced in one of the haplotypes in the panel, while retaining the original sequence of the same haplotype to make the in silico mixture. The other haplotype was excluded from the panel.

To gain an insight into how SNPs that are private to one of the haplotypes (i.e., a variant found only in one population sample but absent in the reference panel) affected haplotype estimation, the proportion of errors that were linked to private sites was recorded for panels 2 and 4. These proportions were then compared to see if there was a significant difference between them using the two-tailed, two-sample test for equality of proportions with the base R function *prop.test()*. Additionally, to explore the relationships between the phasing accuracy and the relatedness between the two mixed haplotypes and between the mixed haplotypes and the reference panel, Hamming distances were calculated (as proxies for genetic distances). This information was combined with the mixture deconvolution results generated using panel 4 to gain insight on how it affected phasing accuracy. To analyze the relationship between the two categories of Hamming distances and the haplotype estimation accuracy, a simple linear regression model was fit to the data. All regression analyses were performed using the *lm ()* function in base R, using the following formula—A ~ M + P, where A is the predicted variable (deconvolution accuracy within each ratio category), and M and P are the predictor variables (the Hamming distance between mixed haplotypes and the minimum Hamming distance between the mixture and the reference panel, respectively). The significance level for all statistical analyses in this study was set at 0.05. 

The 10 haplotype samples were taken two at a time, resulting in 45 distinct combinations of two-person mixtures. This pairing was done for each of six ratios (50:1, 19:1, 9:1, 4:1, 2:1, and 1:1), for a total of 270 separate mixtures. An additional 22 haplotypes from the same database [21] were used to create a reference panel (for a total of 32 haplotypes), which was provided to the software as a prior for haplotypes present in a given sample. The read count was set to 100× based on the suggestion of the algorithm’s author (Zhu personal communication), the number of MCMC steps was set to 3000, the recombination rate was set to 0.0, and all other parameters were set to default values. Although the program has the capacity to estimate as many as five haplotypes, the parameter (k) was constrained to two haplotypes to provide a more simplified scenario. To assess whether the MCMC chain had mixed well, and therefore had converged to the target posterior distribution, trace plots from the runs were visually examined. Trace plots are line charts that display the number of steps on the x-axis and log-likelihood values from a specific draw on the y-axis. An analysis that has converged exhibits a trace plot that explores the sample space widely while still being centered on a single average value, e.g., not showing a long-term trend after an initial transient burn-in phase, traditionally described as a “fuzzy caterpillar” in Bayesian analyses [69,70].

#### 2.1.3. Optimized Reference Panel and Phasing Parameters

Multiple factors are known to affect the accuracy of statistical phasing. They include population genetic features such as the size of the sample, the extent of relatedness between samples and alleles, and population haplotype frequencies [39,71], as well as computational factors such as the number of MCMC steps and haplotype read depth, amongst others. Based on the biological factors, it was projected that panels 2 and 3 would result in lower haplotype accuracy relative to the remaining panels. In those instances, the algorithm would not encounter any rare or private SNPs in the panel that might be present in the mixed individuals, and therefore have no information on LD between the private SNP and other substitutions, although it is worth noting that the lack of LD may be tolerable when there is sufficient quantitation to deconvolve the mixture (e.g., 4:1 mixtures). On the other hand, panels 1 and 4 would allow the algorithm to account for all the sites in the sequence, as they contain either the exact (Panel 1) or the “parent” (Panel 4) sequence of the mixed haplotypes [47]. Consequently, either increasing the size of the panel or including sequences that are as closely related to the mixed haplotypes is a good strategy for improving the accuracy of phasing [47,65,66]. However, given that DEploid’s base algorithm scales quadratically with the size of the reference panel [48], increasing the number of reference sequences can also be computationally taxing and impractical. Accordingly, the preferable tactic, striking a balance between accuracy and functionality, would be to first access a large dataset of haplotypes (to increase the probability of finding related haplotypes) and then filter out a subset of the most closely related haplotypes to comprise the reference panel [39,40,47,48,72].

Likewise, the optimization of analytical parameters is also necessary to achieve high phasing accuracy [39,47]. For example, in a 4:1 ratio (where there is enough quantitative data to resolve donors), a read depth of 100× would mean that a mere 20 reads are available for the minor contributor, whereas that same read-count would amount to 2000× for a read depth of 10,000×. The greater amount of information the latter provides can make a significant difference in the confidence ascribed to haplotype estimation. In the case of DEploid, pilot simulations and correspondence with the author both indicated that coverage of above 100× results in particularly stiff priors. Such priors, which are minimally responsive to the Bayesian updating, result in the reference panel “bleeding” into the estimation of the haplotypes, countering the ability of the estimated sequences to be different than the haplotypes provided in the reference panel. Similarly, considering that this Bayesian algorithm employs a randomized estimation approach, which improves with time [48], the number of MCMC steps set at the beginning of the analysis can dictate whether or not the model has converged on the target distribution or is instead stuck in a local maxima of the posterior [52]. The ideal number of MCMC steps typically varies from one dataset to the other, depending on the dataset’s size, amongst other factors. Early simulations indicated that the default 800 MCMC steps in DEploid were not sufficient for achieving stationarity (i.e., convergence on the posterior distribution). Therefore, the influence of 3000 steps and above was examined regarding the phasing accuracy. The last variable that was manipulated was the graph edit distance. This distance represents the number of changes in the database sequence for it to be compatible with the mixed haplotypes. Varying the graph edit distance results in a change in the size of the database and the average genetic distance between the haplotypes in the database and the mixture. The graph edit distance can, therefore, allow an assessment of the influence of reference panels of varying genetic affinities to the mixed haplotypes. For example, a reference panel with an edit distance of 2 would contain fewer haplotypes than one with an edit distance of 4, but those haplotypes would overall be more closely related to the haplotypes in the mixture than the larger database resulting from the edit distance of 4.

**Figure 2:**
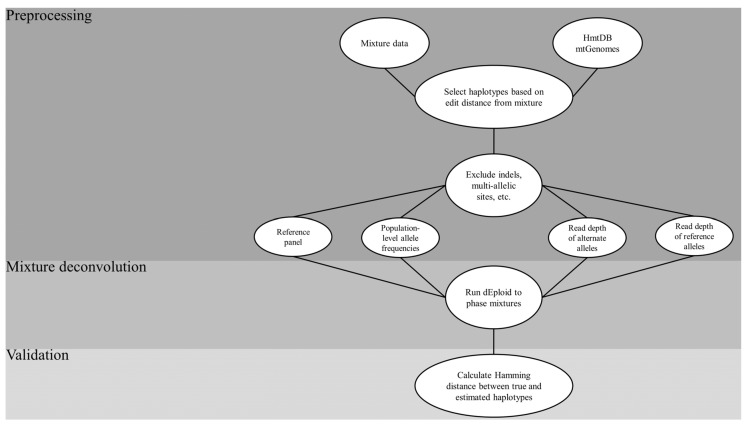
Flow chart of the general process employed in the mixture deconvolution simulation. HmtDB = Human Mitochondrial DataBase; mtGenomes = mitochondrial genomes; indels = insertions-deletions.

Based on the reasoning laid out above, the following steps were implemented to optimize the mixture deconvolution setup (Figure 2):

- Sourcing of the HmtDB for complete mtGenomes;
- Creating in silico mixtures from a sample dataset;
- Selecting haplotypes from the sourced genomes that are a pre-defined edit distance from the haplotypes in the mixtures to create a reference panel;
- Running the core DEploid algorithm to deconvolve the mixtures, using the panel as a prior while tweaking key parameter combinations;
- Assessing the accuracy of the estimated haplotypes that resulted from each combination using the Hamming distance between them.

The wealth of mtGenome data from HmtDB (as described in Section 2.1.1.) was used to increase the diversity of the panel file. The EMPOP files were used to make in silico mixtures that were then deconvolved using the bioinformatic pipeline. Mixture deconvolution simulations were simultaneously configured to investigate the effects of varying parameter values on phasing accuracy. Three separate values for selected variables were chosen for simulations, namely read counts of 50×, 75×, and 100×; MCMC steps of 3000, 6000, and 9000; and graph edit distances of 1, 2, and 4. Since the number of individuals in the panel to a great extent dictates the quality of the phasing, while the runtime increases as the reference panel increases, the panel was restricted to 25 haplotypes that were closest to the mixture. To assess whether the parameters that led to the highest accuracy of haplotype estimation also accurately estimated mixture proportions, the observed proportions of the minor contributor were compared to the expected proportions. To this end, variances in the estimated ratios were compared using the *F*-test for equality of variance. The coefficient of determination values for each of the parameters being tested were also calculated. Both statistical analyses were computed using base R, namely the *var.test ()* and *lm ()* functions, respectively.

Finally, given the nature of the phasing algorithm, three different types of errors were expected:

1. The coercion of a He site into an HoR or HoA site;
2. The coercion of an HoR or HoA site into a He site;
3. Point-switching of alleles between the two haplotypes (e.g., two switches within three consecutive heterozygous sites [42]).

The first two are more likely to occur in imbalanced mixtures, where the software lacks sufficient quantitative information on the minor haplotype and defaults to forcing its alleles to be identical to its major homologue [48]. This effect especially holds true if the position in the minor haplotype has an alternative SNP and the major has the reference allele. On the other hand, the program is reported to have a propensity towards switch errors (i.e., false recombination events in the inferred haplotypes compared with the true haplotypes) when balanced (i.e., 1:1) mixtures are involved [48]. Therefore, the algorithm is more likely to correctly estimate the structure of the major haplotype in imbalanced mixtures than it is in balanced ones. This situation provides a good prospect to interpolate the true sequence of the minor contributor as in a two-person mixture; once a set of alleles has been assigned to the major contributor with certainty, the remaining alleles necessarily belong to the minor contributor. Consequently, all minor sequences for deconvolutions that had accurately phased the major haplotype were re-assessed in the context of the remaining alleles in order to recreate their sequence.

The same 10 haplotypes that were used earlier were combined pairwise to make 45 different two-person mixtures. This pairing was done for each of the six mixture ratios used earlier, coupled with the combination of values for the number of MCMC steps, read depth, and edit distance, producing a final dataset of 7290 (45 × 162 unique parameter combinations) distinct in silico mixtures. The recombination rate was set to 0.0, k was set to 2, and all other parameters were set to default values. The convergence of runs was assessed by visual inspection of trace files. The final output of the simulations documented the number of genomic donors in the mixture, their relative proportions, the phased haplotypes, and the mixture deconvolution accuracy.

### 2.2. Validation with In Vitro Mitochondrial Mixtures

To validate the performance of the pipeline with real mixtures, a set of in vitro mitochondrial mixtures (*n* = 6) was deconvolved. These samples were comprised of 2 two-person mixtures prepared in different ratios (based on quantitation of nuclear markers) of major and minor contributors. More specifically, three mixtures with ratios of 1:50, 1:4, and 1:1 were generated empirically and three other mixtures with ratios of 10:1, 5:1, and 1:1 were taken from Churchill et al., 2018 [3] to allow comparisons of our results against those of the aforementioned study, which deconvolved these same mixtures manually. It is important to note that the 1:1 mixtures in this dataset could be dichotomized into minor and major due to using a nuclear quantification method in this study. This limitation implies that the ratios used here are at best approximations, since it is known that mitochondrial copy numbers can vary from individual to individual [3,16].

The policies and procedures approved by the Institutional Review Board of the University of North Texas Health Science Center in Fort Worth, TX, were followed for the collection and use of samples included in these in vitro mixtures. Samples were quantified with a ThermoFisher Quantifiler Trio DNA Quantification Kit and diluted to appropriate concentrations for two-person mixtures of 100 pg in the aforementioned ratios. The samples were amplified with the ThermoFisher Precision ID mtDNA Whole-Genome Panel Kit, a dual panel pool system that generates 162 short, overlapping amplicons that cover the entire mtDNA genome. Each pool contains 81 primer pairs, as well as degenerate primers to ensure amplification of samples with SNPs in the primer binding region. The 2-in1 method for low copy number samples was followed. Each sample was amplified separately with each set of primers, and then 10 uL from each was combined into a new pool and amplified. Primer sequences were digested, adapters and barcodes were ligated, and samples were subsequently purified with AMPure XP beads. Samples were then quantified by qPCR (Ion Library TaqMan Quantitation Kit) and diluted to 30 pM before being pooled in equimolar fashion. Lastly, libraries were templated on the Ion Chef instrument following the manufacturer’s recommended protocols prior to being sequenced on the Ion S5 (ThermoFisher Scientific).

The sequence data for each mixture were entered into our software in the EMPOP file format alongside the mpileup data, which contained information on variant ratios, as reported by the Converge software [60,73]. The dataset of mtGenomes curated on HmtDB was modified into the reference panel for the analysis in the manner outlined in Section 2.1.1. The mixture deconvolution was run with the read count parameter normalized to 100× and optimized parameters as identified by the simulation results in Section 2.1.3. Finally, the estimated haplotypes were compared to the true haplotypes to assess deconvolution accuracy.

## 3. Results

### 3.1. Differentially Modified Reference Panels

**Figure 3:**
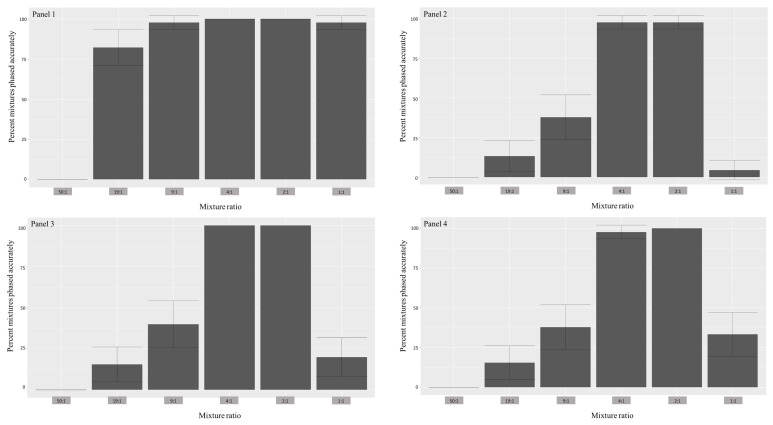
Histograms depicting the percent of mixtures (both minor and major contributors) phased correctly (*y*-axis) using 4 different reference panels for all ratios (*x*-axis). The vertical crossbars represent 95% confidence intervals. For descriptions of panels, refer to the methods section.

As anticipated, reference panel 1 resulted in the highest deconvolution accuracy across all mixture ratios (except the 50:1) compared to other panels (Figure 3). Simulations with panel 3 had deconvolution accuracies that were subequal to panels 2 and 4 in all ratios except for 1:1, where it significantly outperformed them (40% vs. 2% and 20%, respectively). In the context of estimating the major haplotype, all reference panels led to entirely accurate estimations for all ratios, with the exception of the 1:1 mixtures (in the case of 1:1, contributor 1; Table 1). For the 1:1 mixtures, the highest accuracy was observed in reference panel 1 (97.8%), while the poorest was in reference panel 2 (4.4%). In regards to the minor haplotype, reference panel 1 led to the most accurate overall phasing, with 100% accuracy for ratios 4:1 and 2:1 and slightly lower accuracy for 19:1(82.2%), 9:1 (97.8%), and 1:1 (97.8%; in this case, contributor 2). This performance was followed by reference panel 3, which resulted in 100% accuracy for the same ratios as panel 1 but drastically lower percentage of accurate phasing for ratios 19:1 (15.5%), 9:1 (40%), and 1:1 (20%). Reference panels 2 and 4 had the lowest minor haplotype estimation accuracies, with panel 4 resulting in 100% accuracy for only ratio 2:1 and panel 2 with no ratios estimated accurately. Overall, all reference panels performed better in the 4:1 and 2:1 mixture ratios, while struggling the most with the 50:1 and 1:1 ratios. Two-tailed, two-sample *Z*-tests for the equality of proportions showed statistically significant differences between the phasing accuracy levels resulting from the 1:1 ratios for all panel files for the estimation of both haplotypes (Table 2). The same test did not yield significant differences between proportions for the remaining mixture ratios, with one possible explanation being the small sample sizes.

A pronounced statistically significant difference was also observed in the percentage of errors in the phasing values that were associated with private SNPs between panel 2 and panel 3, based on the two-tailed, two-sample *Z*-tests for the equality of proportions (*p*-value = 1.55 × 10^−4^; 95% confidence interval = 0.02–0.08). More specifically, 849 out of a total of 2436 erroneously phased sites were private variants in panel 2, which lacked the sequences closely related to the mitotypes in the mixture. This percentage dropped in panel 3 with the inclusion of haplotypes that were only different by one SNP from one of the mitotypes in the mixture to 633 private sites out of 2141 total errors.

**Figure 4:**
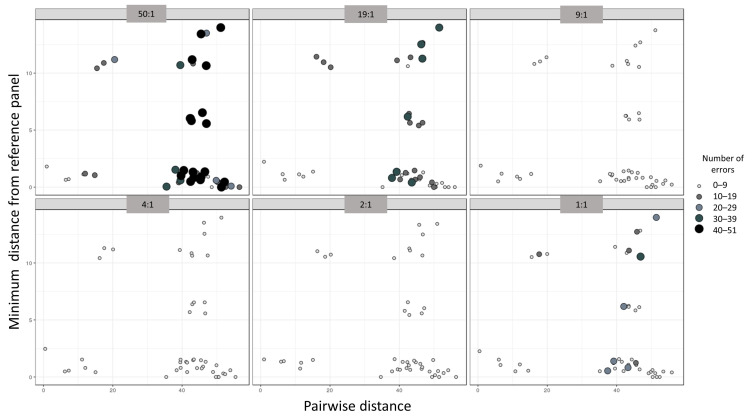
Scatterplot depicting the relationships between the accuracy of phasing in the number of haplotype estimation errors (size and color of the dots), the pairwise distance between the mixed haplotypes (*x*-axis), and the minimum distance from the reference panel (*y*-axis). Each individual panel represents a mixture ratio.

The smallest Hamming distance between individuals in our dataset was 1.0 and the largest was 56.0, with the average being 38.6 (Table S2). The smallest, largest, and average minimum Hamming distances between mixtures and the reference panel were 1.0, 39.0, and 9.7, respectively. The phasing accuracy appeared to be proportional to the pairwise distances in the mixture and inversely proportional to the minimum distance from the reference panel (Figure 4). Linear regression analyses only indicated a statistically significant relationship between the minimum distance from the panel file and the number of phasing errors (*p*-value 6.64 × 10^−4^, Table 3). Overall, the minimum distance from the panel had the largest influence on the 50:1 and 1:1 mixtures and no influence on the 4:1 and 2:1 mixture ratios. 

### 3.2. Enhanced Reference Panel with Fine-Tuned Parameters

**Figure 5:**
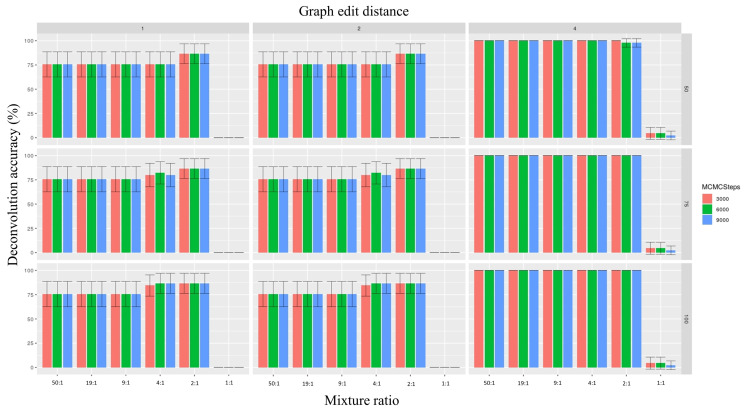
Histograms depicting the percent accuracy of phasing mixtures (both minor and major contributors) correctly resulting from simulations based on different parameter values. The x-axis represents the mixture ratios, the y-axis represents accuracy, and the colors of the bars represent the number of Markov Chain Monte Carlo (MCMC) steps. Each column represents a graph edit distance, while each row represents read counts. The vertical crossbars represent 95% confidence intervals. For descriptions of parameter values, please refer to the Materials and Methods section.

The deconvolution accuracy increased the most noticeably as a function of the graph edit distance. In this context, when the graph edit distance was set to 1, the highest deconvolution accuracy (across all combinations of parameters, number of mixtures = 2430) was 0.0% for the 50:1, 19:1, and 9:1 ratios; 6.8% for the ratio 4:1 ratio; 13.3% for the 2:1 ratio; and 0.0% for the 1:1 ratio. These estimations increased to 75.5% for the 50:1, 19:1, and 9:1 ratios; and to 86.6% for the 4:1 and 2:1 ratios; while remaining at 0.0% for the 1:1 mixtures after the minor mitotype was interpolated from accurately phased major mitotypes (Figure 5). On the other hand, when the graph edit distance was augmented to 4, the deconvolution accuracy was 0.0% for the 50:1 and 19:1 ratios, 2.2% for the 9:1 ratio, 15.8% for 4:1 ratio, 16.7% for the 2:1 ratio, and 0.0% for the 1:1 ratio. After the minor contributor was interpolated from the major contributor, these numbers changed to 100.0% for all mixture ratios, except for the 1:1 mixtures, which increased to 4.4% (Figure 5).

No appreciable change was associated with the adjustment in the read depth or the number of steps within the context of a graph edit distance of 4. Here, for read depths of 50×, 75×, and 100× and numbers of MCMC steps of 3000, 6000, and 9000, the deconvolution accuracy was 100% for all ratios expect for the 1:1 ratio. The 1:1 mixtures had estimation accuracy values of 4.4% for 3000 and 6000 MCMC steps and 2.2% for 9000 steps for all three read depths (Figure 5).

**Figure 6:**
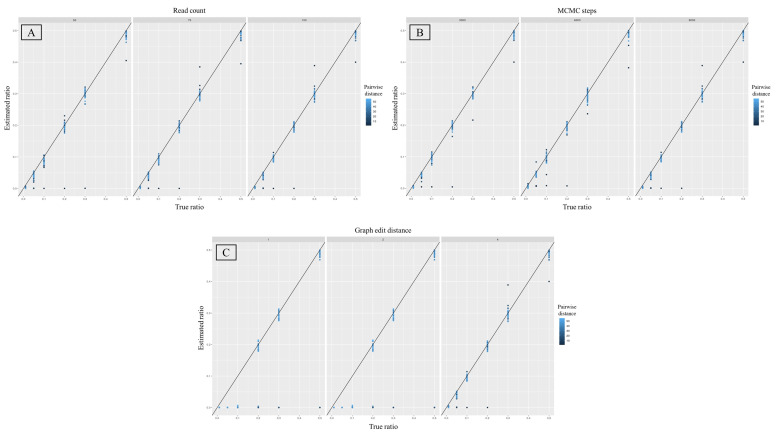
Scatter plots depicting estimated mixture ratios (x axis) against their predicted values (y axis) for six different ratios as they related to each tested parameter, namely read count (**A**), number of MCMC steps (**B**), and the graph edit distance (**C**). Each subplot represents a specific value for the chosen parameter, namely 50, 75, and 100 for (**A**), 3000, 6000, and 9000 for (**B**), and 1, 2, and 4 for (**C**). The color represents pairwise distances, ranging from 10–50, between haplotypes in each mixture.

The accuracy of estimated mixture proportions was also more heavily influenced by changes in the graph edit distances compared to different read depth and MCMC step values (Figure 6). The largest coefficient of determination was associated with a graph edit distance of 4 (R^2^ = 0.99), while the same parameter for the remaining two graph edit distances was much lower (R^2^ = 0.66; Table 4). The coefficient of determination was the same across all three values for both the read depth and number of MCMC steps (R^2^ = 0.74, Table 4). A graph edit distance of 4 also produced the lowest variance in the estimated proportions. The F-test for the equality of variances showed statistically significant differences in the variance of estimated minor proportions (for all mixture ratios combined) between graph edit distances of 1 and 4 and 2 and 4 (*p*-value = 0.003, Table 5). When the graph edit distance was fixed to 4, the variance in the observed minor proportions was the smallest in the 50:1 ratio (0.00001, sd = 0.003), followed by the 19:1 ratio (0.0001, sd = 0.01), the 2:1 ratio (0.0002, sd = 0.016), the 9:1 and 1:1 ratios (0.0003, sd = 0.018), and finally the highest variance was in the 4:1 ratio (0.0008, sd = 0.018). It is useful to note that the majority of outliers in this context were mixtures between highly similar individuals (Figure 6).

### 3.3. Performance with In Vitro Mixtures

The major contributors in all three mixtures generated by [3] were accurately reconstructed, while the minor was estimated accurately only for the 5:1 ratio. Notably, all the sites that were “erroneously” phased were either private point heteroplasmies (PHPs) in one of the contributors or allelic drop-out in the mixed sequence data. PHPs and missing data in general are known to confound mixture deconvolution (regardless of the approach), as currently they are not modeled well. The relative mixture proportions were very closely approximated by DEploid for all three mixtures as $\sim \frac{10}{11},\;\sim \frac{5}{6}$, and $\sim \frac{1}{2}$ for mixture ratios of 10:1, 5:1, and 1:1, respectively.

In the context of mtDNA mixtures generated specifically for this study, the major contributor was estimated correctly based on the read depth for two (50:1 and 4:1) of the three mixtures, while the minor contributor was not estimated correctly in any instance. As in the case of deconvolution of mixtures from [3], all of the errors were either associated with private PHPs, drop-out, or on one occasion with a site in the 4:1 mixture that had near-equal proportions (i.e., $\sim \frac{1}{2}$ instead of $\sim \frac{4}{5}$). The relative mixture proportions were reasonably approximated as $\sim \frac{50}{51},\;\sim \frac{4}{5}$, and $\sim \frac{1}{2}$ for mixture ratios of 50:1, 4:1, and 1:1, respectively.

## 4. Discussion

To the best of our knowledge, only two bioinformatic approaches are currently available for interpreting mtDNA mixtures [10,74]. Both of these algorithms depend on information available from the public mtDNA database Phylotree to assign haplogroups using the EM framework. Nonetheless, both leverage different types of information, with mixemt [10] using the frequency of haplogroups, while the *JaccardBF/ABPI* method [74] relies on matching haplogroup specific variants to assign haplogroups. Despite the fact that both have a high haplogroup assignment accuracy, in our opinion their inability to estimate complete haplotypes restricts their utility for human identification purposes. Moreover, as mentioned earlier, the practice of using reads as working units can result in the introduction of NUMTs and other confounding features. This study proposes a different strategy to deconvolve mtDNA mixtures, i.e., using an MCMC alternative along the lines of statistical phasing on whole mtGenomes. Our method shows high haplotype estimation accuracy across a range of mixture ratios and is relatively fast; for example, a two-person mixture deconvolution with our program involving 3000 MCMC steps and a reference panel of 25 individuals only takes ~9 min. Given that most of our simulations comfortably reached convergence even at 1000 MCMC steps, we estimate that most analyses with a 25-individual reference panel should take anywhere between ~3 and ~9 min. It is also important to note that although the current study only investigates two-person mixtures, it should also be theoretically possible for our method to deconvolve mixtures containing as many as 5 contributors.

Several theoretic and practical factors need to be considered to successfully interpret mixed mtDNA samples. The primary advantage of our method stems from its sourcing of haplotype information from a high-density sequence database. The availability of thousands of mtGenomes from major populations of the world provides information at a finer population genetic resolution (as opposed to the coarser phylogenetic scale), thereby increasing the probability of capturing diagnostic private and rare variants. In this context, our results clearly corroborate the importance of increasing sample size through use of a reference panel of closely related haplotypes [39,40]. Therefore, amongst all parameters that were investigated, it was expected that the ideal graph edit distance was the one with the largest influence on the accuracy of both estimated haplotypes and mixture proportions. A graph edit distance of 4 in the case of our dataset facilitated the inclusion of multiple sequences from HmtDB that were closely related to the haplotypes in the mixture, which was reflected in the significantly higher phasing or deconvolution accuracy across all ratios.

This parameter’s significance increases when it comes to 1:1 ratios, since quantitative data do little to help with the deconvolution when homologous alleles occur in equal frequencies. The relatively poor phasing performance of the 1:1 mixture ratios was attributed to the practical constraint of limiting the reference panel to 25 haplotypes. While this sample size sufficed for the remaining mixture proportions, given the strong haplotype signal in their imbalanced ratios, a larger panel file (or alternatively maybe a higher graph edit distance) is likely to ameliorate the phasing inaccuracy of 1:1 mixtures. The results also show that to a certain extent, mixtures comprised of highly divergent haplotypes, in addition to the small minimum distance from the reference panel, are the most likely to be phased accurately (Figure 4). 

On the other hand, the key to accurately deconvolving highly imbalanced mixtures (e.g., 50:1) probably lies with the user in finding the right balance between increasing the read depth above 100× while simultaneously also increasing the mis-copying rate parameter, which allows the estimated mitotypes to differ from the sequences in the reference panel. This approach would counter the effects of uninformative priors by tolerating greater deviation of the posterior from the prior information provided in the panel. Notwithstanding, at the very least our bioinformatic tool accurately estimates the major haplotype 100% of the time under certain parameters (i.e., graph edit distance of 4) based on our simulated data. This result was largely true even with the deconvolution of in vitro mixtures, given that the errors found in the estimation of the major donor were associated with private PHPs or allelic drop-out rather than misassignment of an allelic state on the part of the algorithm. Thus, in cases with a minor contributor, the user could choose to interpolate the sequence (as outlined in Section 2.1.3), because the results support a successful estimate of the minor contributor 100% of the time. 

When compared to the mixture deconvolution results from [3], our method was comparable to the same in vitro samples in most instances and showed improvements in some cases. For example, He sites that had near-equal relative allele frequencies in the 1:1 mixture (e.g., sites 4086 and 4092) could not be assigned with confidence by [3] until supplementary phylogenetic data were considered, but were parsed accurately by our method. Nonetheless, given the common incidence of personal PHPs, SNPs in the primer binding region, and other interferences, variance in the relative proportion of sites within a given sample can diminish accuracy. This issue applies to our analytical framework as well, especially if the variance is very pronounced. Our method was also able to phase both donors in the 5:1 mixture, while the minor haplotype in the same sample was only partially reconstructed by the approach employed in [3]. On the other hand, our technique fared as poorly as [3] when it came to coping with private PHPs, for the same reasons outlined by that study. All things considered, the primary advantage of our method lies in being able to automate mixture deconvolution, which is otherwise a cumbersome manual process.

While our method is automated in general, the user still needs to be mindful of several assumptions related to the input data and the ramifications of instances when these assumptions are violated (see Table S3). The most important of these assumptions is probably the implicit expectation that the mixed mtDNA data considered are correctly genotyped [39,57,75]. At the very least, the user should assess the information on variant frequencies and calls produced by the variant caller (e.g., Converge), as well as the actual BAM file. This review will allow verification that all He sites have been called correctly (since the genotyping software is prone to errors in this context). Moreover, given that the decision of the software to recognize the alternate allele at any site is dependent on the threshold that has been set to call PHPs, the user may need to factor the thresholding while interpreting the results. Similarly, it would be prudent for the user to look at the table of alternate and reference allele counts and identify heterozygous sites that are incongruent in their relative proportions in comparison to other sites in the mixture; as demonstrated by our deconvolution of in vitro mixtures, including those sites could impact the algorithm’s ability to estimate the true haplotype. Regardless, the ability to reconstruct even partial mitotypes is valuable, since current forensic profiling approaches do allow missed sites and the accuracy of haplotype estimation does not need to be 100%.

As with the phasing accuracy, the accuracy of the estimated mixture proportions appeared to be closely related to the graph edit distance, again underscoring the need for an appropriate panel file. However, the pairwise genetic distance between haplotypes in the mixture seemed to have a larger contribution in this context, given that the majority of erroneous observed proportions were associated with mixtures involving closely related individuals. For example, most outliers were between two of the three haplotypes (NaBr05, NaBr07) that belong to the same haplogroup and were only two SNPs apart. The third haplotype (NaBr09) in that haplogroup was 5 and 4 SNPs away from NaBr07 and NaBr05, respectively. Reassuringly, even though the proportions were not accurately calculated, the algorithm was able to correctly estimate the haplotypes involved in these mixtures. 

Several shortcomings and limitations still persist with our method, the most acute of which is the quadratic increase in computation time that is associated with increasing the size of the reference panel. The issue of runtime is especially important, as sample size is the most critical parameter for accurate phasing (i.e., the quality of the reference panel). However, with advances in high-throughput technology, concomitant with the reduction in sequencing costs, sample sizes will likely become a transient issue. This should result in larger databases, which will permit the inclusion of mitotypes that are even more similar to a mixture, and in doing so should improve the quality of the resultant deconvolved haplotypes. Additionally, DEploid, the core algorithm in our phasing pipeline, currently does not accommodate indels or multi-allelic sites, which could be good sources of diagnostic variation [39,48]. It is to be noted though that the requirement for bi-allelic SNPs is a guideline and not a strict requirement for the algorithm (i.e., multi-allelic sites can be split as the per bcftools norm [76], and while not recommended by DEploid, the encoding style permits the inclusion of indels). Also important to note is that the phasing accuracy is dependent not just the on the quantity but on the quality of the reference panel [66]. While users can address the former by increasing the size of the panel, assuring that the reference haplotypes are themselves accurate is not something they may readily control. Finally, no matter how sophisticated the algorithm, statistical phasing will always struggle with rare variants such as de novo mutations in individuals, and in this context experimental phasing methods might be the more effective alternative [39].

Despite these limitations, our approach is the only bioinformatic tool that automates the deconvolution of mixed mtDNA profiles into complete haplotypes (as opposed to haplogroups). Moreover, our pipeline is also the only available open source continuous software in this context. The mixture deconvolution method presented herein, thus, represents a substantial step forward in the analysis and interpretation of mixed mtDNA profiles, a process that has been historically difficult within the field of applied forensic genetics.

## Figures and Tables

**Table 1:** Phasing accuracy as it relates to different reference panels for all mixture ratios. The values in parentheses are standard deviations.

	Reference Panel 1		Reference Panel 2		Reference Panel 3		Reference Panel 4	
Proportions	Major	Minor	Major	Minor	Major	Minor	Major	Minor
50:1	100%	0%	100%	0%	100%	0%	100%	2.2% (4.3%)
19:1	100%	82.2% (11.8%)	100%	13.3% (9.9%)	100%	15.5% (10.6%)	100%	20% (11.7%)
9:1	100%	97.8% (4.3%)	100%	37.8% (14.1%)	100%	40% (14.3%)	100%	35.5% (14%)
4:1	100%	100%	100%	97.8% (4.3%)	100%	100%	100%	97.8% (4.3%)
2:1	100%	100%	100%	97.8% (4.3%)	100%	100%	100%	100%
1:1	97.8% (4.3%)	97.8% (4.3%)	6.6% (7.3%)	4.4% (6%)	20% (11.7%)	20% (11.7)	40.9% (14.36%)	38.6% (14.2%)

**Table 2:** Results of the two-tailed Z-tests for the equality of proportions, testing for statistically significant differences in the number of haplotypes phased correctly between each panel in the context of 1:1 mixture ratios.

	Major (Panel 3)	Major (Panel 4)	Minor (Panel 3)	Minor (Panel 4)	Major (Panel 3)	Major (Panel 1)	Minor (Panel 3)	Minor (Panel 1)	Major (Panel 3)	Major (Panel 2)	Minor (Panel 3)	Minor (Panel 2)
Number of successes	19	9	18	9	19	44	18	44	19	3	18	3
95% confidence interval	0.014–0.430		0.007–0.407		0.383–0.728		0.406–0.749		0.172–0.539		0.178–0.533	
*p*-value	0.04		0.06		3.38 × 10^−8^		1.254 × 10^−8^		0.0002		0.0001	

**Table 3:** The linear regression results, testing for statistically significant effects of the minimum distance from the reference panel and the pairwise distance of mixed haplotypes on deconvolution accuracy.

	Estimate	Standard Error	*t*-Value	*p*-Value for *t*-Test
Minimum Distance from Panel	−2.2381	0.6497	−3.445	0.000664
Pairwise Distance	−0.1939	0.2082	0.931	0.352452

**Table 4:** Comparing coefficients of determination for different parameters as they relate to the goodness of fit of the relationship between expected and observed mixture proportions.

Read Count		MCMC Steps		Graph Edit Distance	
50	R^2^ = 0.74	3000	R^2^ = 0.74	1	R^2^ = 0.66
75	R^2^ = 0.74	6000	R^2^ = 0.74	2	R^2^ = 0.66
100	R^2^ = 0.73	9000	R^2^ = 0.74	4	R^2^ = 0.99

**Table 5:** Results of the F-test for the equality of variance of the estimated mixture proportions between different values of the graph edit distances.

Graph Edit Distance	1 vs. 2	1 vs. 4	2 vs. 4
95% confidence interval	0.924–1.083	1.039–1.218	1.039–1.218
*p*-value	1.0	0.003	0.003

## Supplementary Materials

The following are available online at https://www.mdpi.com/2073-4425/12/2/128/s1: Table S1: Information on the population, sample name, and haplogroup for the individuals used to generate the mixtures in this study. Table S2: Hamming distances within and between haplotypes used in this study. Table S3: List of assumptions involved in the deconvolution of mtDNA mixtures using the proposed method and ramifications when these assumptions are violated.
