# Supplementary material for: A Continuous Statistical Phasing Framework for the Analysis of Forensic Mitochondrial DNA Mixtures
Source: Genes (Basel). 2021 Jan 20;12(2):128. doi: 10.3390/genes12020128 (PMC7909279; doi:10.3390/genes12020128)
Supplement: Supplementary file 1 [file genes-12-00128-s001.pdf]

Table S1. Information on population, sample name and haplogroup for the individuals used to generate the mixtures in this study (from Strobl et. al.2019).

Population	Sample Name	Haplogroup
Brazilian Native American	NaBr02	B2e
Brazilian Native American	NaBr04	A2+(64)
Brazilian Native American	NaBr05	B2e
Brazilian Native American	NaBr06	D1
Brazilian Native American	NaBr07	B2e
Brazilian Native American	NaBr08	B+C17:C572
Brazilian Native American	NaBr09	A2ac
Brazilian Native American	NaBr10	K1a4a1
Brazilian Native American	NaBr11	C1b
Brazilian Native American	NaBr12	A2+(64)+16129

Table S2. Hamming distances within and between haplotypes used in this study.

Minimum pairwise distance	1.0
Maximum pairwise distance	56.0
Average pairwise distance	38.6
Smallest minimum distance from the panel	1.0
Largest minimum distance from the panel	39.0
Average minimum distance from the panel	9.7

Table S3. List of assumptions involved in the deconvolution of mtDNA mixtures using the proposed method and ramifications when these assumptions are violated. He = one copy of the alternative (i.e. non reference) allele and one of the reference; HoR = two copies of the reference nucleotide

Assumption	Result of violation
No allelic drop-outs	The algorithm lacks information to assign dropped sites to phased haplotypes
No point heteroplasmies	The algorithm duplicates the heteroplasmic position in the phased haplotype
All He sites called	The algorithm falsely calls the position a HoR site
Relative proportions of allele approximately constant across all sites (i.e. low variance)	The algorithm lacks information to assign sites that deviate from other sits in relative proportion
